# Supplementary material for: Dynalign II: common secondary structure prediction for RNA homologs with domain insertions
Source: Nucleic Acids Res. 2014 Nov 21;42(22):13939–48. doi: 10.1093/nar/gku1172 (PMC4267632; doi:10.1093/nar/gku1172)
Supplement: SUPPLEMENTARY DATA [file supp_gku1172_nar-02021-z-2014-File012.zip › manual/Overview/Running_Java_GUI.html]

RNAstructure Installation and Overview: Running the JAVA GUI Interface


|  |  |  |
| --- | --- | --- |
|  | RNAstructure Installation and Overview Running the JAVA GUI Interface | - Contents - Index |
| General Details  The RNAstructure JAVA GUI interface is a cross-platform user-freindly front-end for RNAstructure. The backend functions are the same C++ as the rest of the package to facilitate speed. The backend is connected to the GUI using SWIG.  After the JAVA GUI is built (or the executable downloaded), it should be run using one of two scripts in the RNAstructure/exe directory.  On Unix/Linux/OS X systems, the GUI can be started by running the RNAstructureScript script file.  On DOS/Windows systems, the GUI can be started by running the RNAstructure.bat script file.  Note that one should NOT attempt to run the RNAstructure.jar (JAVA JAR archive) or RNAstructure library (name depends on the operating system, see the "Libraries" page for details) file to open the GUI. These files work together, and the scripts above handle their proper integration. | | |
| Visit The Mathews Lab RNAstructure Page for updates and latest information. | | |
